# Transform diabetes care with precision medicine

**DOI:** 10.1002/hsr2.1642

**Published:** 2023-10-30

**Authors:** Sharumathy Kannan, Dinesh Kumar Chellappan, Chia Siang Kow, Dinesh Sangarran Ramachandram, Manisha Pandey, Jayashree Mayuren, Kamal Dua, Mayuren Candasamy

**Affiliations:** ^1^ School of Health Sciences International Medical University Kuala Lumpur Malaysia; ^2^ Department of Life Sciences, School of Pharmacy International Medical University Kuala Lumpur Malaysia; ^3^ Department of Pharmacy Practice, School of Pharmacy International Medical University Kuala Lumpur Malaysia; ^4^ School of Pharmacy Monash University Malaysia Subang Jaya Selangor Malaysia; ^5^ Department of Pharmaceutical Sciences Central University of Haryana Mahendergarh India; ^6^ Department of Pharmaceutical Technology, School of Pharmacy International Medical University Kuala Lumpur Wilayah Persekutuan Malaysia; ^7^ Faculty of Health, Australian Research Centre in Complementary and Integrative Medicine University of Technology Sydney Ultimo New South Wales Australia; ^8^ Discipline of Pharmacy, Graduate School of Health University of Technology Sydney Ultimo New South Wales Australia

**Keywords:** artificial intelligence, beta cell transplantation, diabetes, diabetes nanomedicine, precision medicine

## Abstract

**Background and Aims:**

Diabetes is a global concern. This article took a closer look at diabetes and precision medicine.

**Methods:**

A literature search of studies related to the use of precision medicine in diabetes care was conducted in various databases (PubMed, Google Scholar, and Scopus).

**Results:**

Precision medicine encompasses the integration of a wide array of personal data, including clinical, lifestyle, genetic, and various biomarker information. Its goal is to facilitate tailored treatment approaches using contemporary diagnostic and therapeutic techniques that specifically target patients based on their genetic makeup, molecular markers, phenotypic traits, or psychosocial characteristics. This article not only highlights significant advancements but also addresses key challenges, particularly focusing on the technologies that contribute to the realization of personalized and precise diabetes care.

**Conclusion:**

For the successful implementation of precision diabetes medicine, collaboration and coordination among multiple stakeholders are crucial.

## 1 INTRODUCTION

Diabetes is a global health concern; 537 million adults are living with diabetes in 2021 and this number is predicted to rise to 643 million by 2030 and 783 million by 2045.^1^ According to the statistics provided by the International Diabetes Federation, in 2021, approximately 6.7 million adults died as a result of diabetes or its complications.^2^ This corresponds to 12.2% of global deaths from all causes.^2^ Without proper interventions, it is predicted that by 2035, 592 million people will die due to diabetes.^3^ Currently, at least 79% of individuals who are diagnosed with diabetes live in low‐ or middle‐income countries.^4^, ^5^, ^6^

In type 1 diabetes mellitus (T1DM), there is an autoimmune response directed against the beta‐cells, leading to the initiation of a complex cascade of proinflammatory reactions. This immune response leads to the destruction of insulin‐producing beta‐cells in the pancreas, resulting in a lack of insulin production and subsequent high blood glucose levels. This process entails the presentation of beta‐cells antigens by the antigen‐presenting cells to the immune system which then produces a cascade of immunological responses that target beta‐cells.^7^ On the other hand, individuals with type 2 diabetes mellitus (T2DM) often exhibit a primary genetic predisposition that results in diminished beta‐cell function. It is noted that the pathophysiological progression of T2DM begins with normal glucose tolerance, transitions to impaired glucose tolerance, and ultimately culminates in overt diabetes due to the concomitant decline in both insulin sensitivity and beta‐cell function.^8^ The pathogenesis of T2DM stems from the impaired action of insulin and secretion of insulin, as well as the endogenous glucose output.^9^, ^10^ An emerging form of diabetes, known as type 3 diabetes, is characterized as a neuroendocrine disorder that signifies the progression of T2DM to Alzheimer's disease. This designation is based on the recent evidence suggesting that patients with T2DM and Alzheimer's Disease share some common characteristics, including the presence of amyloid beta deposits in both the brain and pancreas.^11^

Pharmacological interventions are important for the management of diabetes. Currently, the majority of patients who are diagnosed with T2DM rely on glucose‐lowering drugs to achieve normoglycemia. Metformin is widely used as the first‐line glucose‐lowering drug in patients with T2DM due to its advantages, such as absence of weight gain and a low risk of hypoglycemia. Since T2DM is a progressive disease, many patients will eventually require insulin therapy as the disease advances. This is a consequence of the pancreas losing its ability to produce an adequate amount of insulin over time to meet the body's requirements. Insulin therapy serves the purpose of regulating blood glucose levels and preventing complications associated with diabetes.^12^, ^13^

Glinides represent another conventional therapeutic approach for T2DM. Functioning as insulin secretagogues, they are instrumental in managing postprandial hyperglycemia.^14^ Glucagon‐like peptide‐1 (GLP‐1) and dipeptidyl peptidase‐4 inhibitors (DPP4is) are incretin‐based medications have gained prominence in the management of hyperglycemia in T2DM patients. They work by increasing insulin secretion and promoting glucose uptake in peripheral tissues, while reducing the release of glucagon and slowing down gastric emptying. Incretin hormones, which are peptides released in response to nutrient ingestion, play a pivotal role in stimulating insulin secretion. As a result, incretin‐based therapies have the potential to emerge as frontline treatments for T2DM patients.^15^, ^16^

Precision medicine involves the integration of a broad range of individual data, including clinical, lifestyle, genetic, and other biomarker information. It refers to personalized treatment that utilizes the latest diagnostic and therapeutic technologies to cater to patients' unique genetic make‐up, molecular markers, phenotypic characteristics, or psychosocial factors. By tailoring treatment to the specific needs of each patient, precision medicine offers the potential for more effective and efficient healthcare delivery.^17^ This rapidly advancing field continuously witnesses breakthroughs and innovations. Notably, to facilitate the proper organization of clinical information, electronic health records have been devised to construct a comprehensive database for precision medicine.^18^ If the efforts in precision medicine continue to grow and expand, next‐generation DNA sequencing methods, gene therapy, and genetics could potentially see significant cost reduction in the medical field.^19^ To further improve precision medicine, it is important to invest in the best equipment and cultivate a thorough understanding of disease pathobiology.^20^ In 2018, the Precision Medicine in Diabetes Initiative (PMDI) was launched by the American Diabetes Association and the European Association for the Study of Diabetes (EASD), with the aim of advancing precision diabetes care.^21^

The main difference between precision medicine and standard medical approaches lies in the use of complex data to evaluate a patient's health status, medical history, diagnosis, and anticipated treatment outcomes. Precision medicine can also be used to identify patients who do not require medical attention through the analysis of relevant healthcare data from various sources, including clinical records and big data.^22^ The technological facets of precision medicine has helped to categorize genetic variations among millions of individuals worldwide, consequently revealing hundreds of variants associated with T2DM.^23^

Identifying the underlying cause of a disease is crucial for understanding its pathway and pathogenesis, which in turn facilitates the exploration of precise treatments tailored to specific individuals. This personalized approach to treatment development is advantageous for distinct patient populations, moving away from the “one‐size‐fits‐all” approach commonly used in modern medicine.^24^ Further exploration into genetic analysis can aid in precision medicine by categorizing patients into subgroups with different genetic and molecular compositions. This approach goes beyond categorizing patients into broad classifications, such as T1DM and T2DM. In diabetes, precision medicine can be used to distinguish between T1DM, T2DM, neonatal diabetes, and monogenic diabetes, including maturity‐onset diabetes of the young (MODY).^19^

It is widely appreciated that precision medicine has the potential to improve the prevention, management, and treatment of complex diseases such as diabetes. Its success relies on the integration of genetic information with internal and external environmental factors, which can significantly impact the susceptibility, progression, and phenotype of the disease, particularly in the context of diabetes. Following a diabetes diagnosis, various therapeutic options are available for disease management. These may include blood glucose monitoring, lifestyle interventions, and glucose‐lowering drugs targeting specific disease pathways. However, the essence of precision medicine ilies in tailoring therapies to individual characteristics, aiming for curative outcomes while minimizing side effects.^25^ Different precision medicine approaches have been developed to address distinct types of diabetes. For instance, in the case of T1DM, advancements in glucose monitoring devices, insulin pumps, closed‐loop systems, and artificial pancreas technologies have significantly improved the quality of life for patients with this condition. In contrast, individuals with monogenic diabetes often respond well to sulfonylureas, effectively lowering their blood glucose levels.^26^

The full integration of precision medicine into healthcare can become reality if healthcare providers, employers, and policymakers gain a better understanding of its fundamental concepts. While precision medicine is progressively finding its way into practice, not all patients have had the opportunity to reap its benefits. Undoubtedly, precision medicine is the future of the medical field, promising a significant impact on both behavioral and biomedical research in the centuries to come.^27^ When discussing the use of precision medicine, it is essential to include the different types of omics with clinical applications. Although precision medicine has yet to achieve widespread adoption, it carries the promise of life‐changing impacts, especially for patients grappling with chronic conditions such as diabetes.^28^

## 2 LITERATURE SEARCH

To comprehensively explore the application of precision medicine in both T1DM and T2DM, an extensive literature search was conducted. This search aimed to identify relevant studies, research articles, and scholarly publications that shed light on the evolving landscape of precision medicine in diabetes care. The following sections detail the methodology employed for this literature search.

### 2.1 Databases utilized

The databases PubMed, Google Scholar, and Scopus were selected for their wide coverage of biomedical and clinical literature. PubMed, a prominent resource in the field of medicine, ensured access to peer‐reviewed articles from reputable journals. Google Scholar, with its expansive database, allowed for a more comprehensive exploration of grey literature and emerging research. Scopus, known for its interdisciplinary coverage, facilitated the inclusion of studies from various related fields, enriching the breadth of our review.

### 2.2 Keywords and search terms

The search queries were thoughtfully crafted to capture a wide spectrum of articles pertaining to diabetes and precision medicine. The primary keywords and search terms used were “diabetes,” “precision medicine,” “personalized medicine,” “personalized medicine,” and “hyperglycemia.” These search terms were selected to encompass various facets of diabetes care, genetic considerations, and precision approaches.

### 2.3 Language criteria

In alignment with the accessibility of our review, the literature search was limited to studies published in the English language. This criterion was applied to ensure that the retrieved articles were accessible to a broader readership and to maintain consistency in language throughout our analysis.

### 2.4 Search strategy

The literature search was conducted using a combination of the specified keywords and search terms. A systematic approach was employed to retrieve relevant articles, encompassing both established databases and newer sources to ensure a comprehensive search. This search strategy allowed us to identify the latest research findings, technological advancements, and clinical applications of precision medicine in diabetes management.

Overall, our rigorous literature search and selection criteria were designed to provide a robust foundation for our review, enabling us to present a current and informed analysis of precision medicine's role in diabetes care.

## 3 PATHOPHYSIOLOGY OF DIABETES

### 3.1 T2DM

The pathophysiology of T2DM is characterized by impaired insulin secretion and insulin resistance. Insulin is a protein produced by pancreatic beta‐cells in response to elevated glucose concentrations. This insulin response unfolds in two phases; the first‐phase insulin response occurs within 2–4 minutes and declines rapidly after 10–15 min, while the second‐phase insulin response is more gradual, reaching steady‐state levels after 2–3 hours. T2DM primarily stems from genetic factors that compromise beta‐cell function. However, these genetic predispositions are often compounded by factors such as obesity and age‐related insulin resistance.^29^ When the feedback loop between insulin action and secretion is impaired, blood glucose levels surge. Moreover, elevated levels of free fatty acids (FFAs), including palmitic acid and stearic acid, increase the risk of developing T2DM by inducing insulin resistance, beta‐cell dysfunction, and hyperglycemia. They also contribute to obesity while triggering mitochondrial dysfunction and oxidative stress. Excess FFAs and hyperglycemia can lead to the impairment of beta‐cells by inducing endoplasmic reticulum (ER) stress through activation of the apoptotic unfolded response (UPR) pathways. The stress induced by excess saturated FFAs activates the UPR pathway, leading to inhibition of the sarco/ER Ca2+ ATPase (SERCA), activation of IP3 receptors, or direct damage of ER homeostasis.^30^ Insulin resistance, marked by heightened glucose production in the liver and reduced glucose utilization in the liver, muscles, and adipose tissue, is a key player in T2DM. While beta‐cell dysfunction carries more severe consequences than insulin resistance, the presence of both factors can exacerbate the progression of T2DM.^9^

Nevertheless, it has also been revealed that the impairment of beta‐cells in T2DM could be due to a much bigger and complex association related to the environment factors and various molecular pathways involved in cell biology.^31^ Overnutrition at a specific stage in a patient's life can result in chronic conditions such as hyperglycemia and hyperlipidemia, often leading to insulin resistance and chronic inflammation. Moreover, the pathophysiology of T2DM can also be influenced by genetic susceptibility differences present in beta‐cells. This intricate interplay of genetic predisposition, environmental factors, and complex molecular pathways highlights the personalized nature of T2DM. The pathophysiology of T2DM can manifest differently in individuals due to their unique genetic susceptibilities and life experiences. Precision medicine in the context of T2DM acknowledges this heterogeneity, seeking to tailor treatments to individual genetic, molecular, and lifestyle profiles. By delving into the specific genetic makeup, phenotypic characteristics, and molecular underpinnings of each patient, precision medicine offers a promising avenue for more effective and efficient healthcare delivery in managing and preventing T2DM. Understanding the multifaceted origins of this disease and its progression provides a solid foundation for the development of tailored therapies, ultimately striving for better outcomes and improved quality of life for those affected by T2DM.

### 3.2 T1DM

The association between the immune system and T1DM was first established in 1973, when a connection between human leukocyte antigens (HLA) and insulin‐dependent diabetes mellitus was discovered.^32^ It has since been established that HLA contribute to at least 50% of the genetic predisposition to T1DM, indicating the selective nature of specific autoantigen peptides in the pathogenesis of the disease.^33^

T1DM is characterized by a high frequency of islet‐specific autoreactive CD8 + T lymphocytes and impaired immune function. Several lines of evidence such as the transfer of T1DM following non‐T‐cell depleted allogeneic bone marrow transplantation, the development of T1DM in an individual with B‐lymphocyte and antibody deficiency, and the identification of inherited genetic defects of T‐lymphocyte function associated with T1DM, highlights the crucial role of T cells in the pathophysiology of the disease.^34^

To ameliorate hyperglycemia, beta‐cells usually undergo partial recovery of insulin secretory function during a period known as the “honeymoon period”. This period allows for limited exogenous insulin use as endogenous insulin production improves. However, over time, the remaining beta‐cells undergo a decline in function and number, though complete depletion of all B cells does not typically occur.^33^ With our deepening understanding of the immune system's intricate role and genetic predisposition in T1DM, precision medicine aims to tailor treatment strategies to individual patients based on their specific immune profiles, genetic markers, and autoantibody patterns. By deciphering the unique genetic and immunological factors contributing to T1DM in each patient, precision medicine opens the door to more personalized and effective interventions. This approach extends beyond traditional "one‐size‐fits‐all" treatments, offering the potential to halt the autoimmune process, preserve beta‐cell function, and optimize therapeutic strategies during the critical honeymoon period. The integration of precision medicine into T1DM management represents a promising avenue for improving long‐term outcomes and enhancing the quality of life for individuals affected by this autoimmune condition.

## 4 PRECISION MEDICINE AND OPPORTUNITY IN DIABETES

Precision medicine is an emerging approach to disease treatment and prevention that takes into account individual variability in genes, environment, and lifestyle. It aims to provide customized strategies for prevention and management, unlike the traditional one‐size‐fits‐all approach, which uses the standardized prevention and treatment regimens for every patient regardless of their response to the therapy. Precision medicine strives to tailor strategies based on the patient's unique genetic and environmental makeup.

Distinguishing between precision medicine and personalized medicine reveals fundamental differences in methodology and healthcare delivery. Personalized medicine is a therapeutic approach that takes into account a patient's genetic makeup, integrating their knowledge, environment, preferences, social context, and other daily life factors into a tailored treatment plan. In contrast, precision medicine extends beyond genomics, anchoring itself in data analytics and information‐driven healthcare models. While personalized medicine primarily revolves around individual patient care within the patient–provider relationship, precision medicine forms an intricate ecosystem that interconnects patients with a broader network encompassing healthcare providers, researchers, and clinical laboratories.^21^ The complexity of precision medicine notwithstanding, it has emerged as a pivotal paradigm in the field of medicine, particularly in enhancing the diagnosis, treatment, and management of chronic diseases like diabetes.

Indeed, the integration of omics profiling technologies, big data, and machine learning in precision medicine for diabetes has the potential to revolutionize diabetes care. By analyzing genetic, transcriptomic, proteomic, and metabolomic data, clinicians can identify distinct subsets of patients with diabetes and tailor treatments that are most effective for each individual.^35^, ^36^ Additionally, precision medicine can help to identify patients who are at higher risk of developing diabetes, enabling early interventions and prevention strategies. Moreover, precision medicine can also address the issue of patient non‐adherence to treatment regimens. By providing patients with personalized treatment plans, based on their individual genetic and environmental factors, they are more likely to adhere to their treatment regimens, and ultimately achieve better clinical outcomes. This in turn, benefits not only the patient but also reduces healthcare costs associated with diabetes management. Overall, the emergence of precision medicine in diabetes care represents a significant advancement in the field of medicine, offering a more individualized and effective approach to diabetes management and treatment. As the technology and accessibility continue to improve, precision medicine has the potential to greatly enhance the lives of individuals grappling with diabetes.^37^

Moreover, the essence of precision medicine revolves around the fine‐tuning of treatment plans at the individual patient level. Even though there is an elaborate understanding of the epidemiology and pathophysiological pathways of diabetes, there has been little progress in terms of diagnosis or selecting the most suitable medications for patients. Currently, medications are prescribed based on population‐wide averages, which unfortunately do not cater to the unique needs of all patient groups. Precision medicine seeks to address this by demarcating different subgroups of diseases and identifying biomarkers that can be optimized treatment outcomes. To personalize the medical profile for patients requiring such an approach, it is important to use traditional sources of medical information such as laboratory workups, patient history, and physical examination. The application of data mining techniques to this information can help to optimize patient care within the realm of precision medicine.^35^ The main objective of precision medicine in the context of diabetes is not only to recognize responders or nonresponders to glucose‐lowering drugs, but to identify those who will potentially benefit the most from a particular medication. This is possible when robust biomarkers are in play, capable of influencing whether an individual exhibits a stronger or weaker response to each drug class. In cases where there are no single markers with substantial effect sizes, the next approach would be to use multiple markers in combination to carefully select treatment options for individuals.^38^

Precision medicine relies on recognizing and combining personal variability in terms of genomics, physiology, biology, and environmental factors to create a “personalized” approach to disease management, prevention, and treatment for each individual. A personalized outlook is currently applied in experimental settings and in conditions where there are unpredictable risks, as disease manifestations and therapy failure can be avoided. Advanced technologies that are becoming more prominent in the field of biomedicine encourage the collection of a large amount of information that can be used to promote accurate and targeted approaches to diagnosis, management, prevention, and therapy. Traditional medical treatments focus on a one‐size‐fits‐all approach, leading to varying biological responses. In stark contrast, precision medicine operates on the premise that healthcare solutions can be better tailored for specific individuals or well‐defined patient subsets.^39^ Precision medicine aims to improve healthcare by prescribing the most effective course of treatment for each individual patient, which ultimately leads to better quality of care.^40^

T1DM is a multifactorial autoimmune disorder characterized by the T‐cell‐mediated destruction of pancreatic beta cells, resulting in insulin insufficiency. Currently, patients with T1DM receive a standardized treatment in the form of exogenous insulin replacement therapy, which often falls short of achieving optimal blood glucose levels in many patients. In the realm of T1DM management, efforts are dedicated to mitigating the risks associated with the development of diabetes, which includes preclinical detection and innovative treatment methods, such as gene therapy. Insulin pumps are becoming increasingly popular in managing T1DM and are gradually replacing insulin injections. These pumps allow patients to control their insulin dosages by administering controlled amounts of insulin subcutaneously, and thus it helps prevent the occurence of both hypoglycemia and hyperglycemia. The effectiveness of this technology can be enhanced when paired with continuous glucose monitoring (CGM) devices, which have been shown to improve blood glucose control and lower the long‐term risks of diabetic complications.^41^ The artificial pancreas approach involves combining CGM with continuous insulin infusion to achieve optimal blood glucose control. When a continuous glucose monitor is combined with a controlled algorithm and an implanted insulin pump, individuals with T1DM can achieve better glycemic outcomes while reducing their reliance on self‐management.^42^ The concept of an artificial pancreas eliminates the need for T1DM patients to manually control their daily insulin and glucagon delivery.

Moreover, T1DM patients who experience hypoglycemic episodes and other complications may benefit from noninsulin antidiabetic drugs through future clinical trials. Notably, medications such as metformin and pramlintide have shown to be effective blood glucose regulators and could play a pivotal role in maintaining glycemic control in T1DM. Moreover, the combination of GLP‐1 agonists with insulin has the potential to reduce the daily insulin dosages, a strategy proven to improve glucose control and promote weight loss.^43^

In contrast to T1DM, T2DM is characterized by multiple low‐impact risk variants, pervasive environmental exposures, and a phenotype that falls on a quantitative spectrum of metabolic disturbances. One of the goals of precision medicine in the clinical management of patients with T2DM is to include genomic, transcriptomic, and metabolic data to predict the development of unwanted complications and enable preventive intervention. The best approach to preventive therapy is to anticipate the response and tolerance of each individual to specific therapies, which allows for the development of effective drug formulations, exercise therapies, and dietary restrictions based on individual responsiveness. In the era of precision medicine, diabetes care envisions that patients and healthcare personnel will utilize decision support systems integrated into electronic medical records, along with the most recent evidence‐based guidelines and literature updates. These systems will provide healthcare personnel with the most up‐to‐date information on the risks of diabetes, which can aid in providing optimal diagnosis, treatment, monitoring, and management plans.^44^ While personalized diabetes care has been introduced for many years, the complexity of implementing these guidelines into the routine management of T2DM remains a challenge.^45^ Recognizing that the therapy and management of T2DM must be personalized according to each individual within the continuum of diabetes, it has been suggested to classify diabetes based on the degree and rate of decline in beta‐cell function, which would revolutionize the personalized therapy of T2DM.

In terms of disease monitoring, there are new technologies that can aid in precision monitoring. These technologies involve a comprehensive evaluation of biological indicators, such as the utilization of CGM, as well as monitoring lifestyle behaviors like physical activity, dietary habits, stress levels, and sleep patterns. Digital applications, sensor technologies like robot sensors and ingestible sensors, as well as blood assays can all be used to achieve precision monitoring. In fact, precision monitoring lays the foundation for precision prevention. For example, in T1DM, closed‐loop systems that use glucose sensing technologies and algorithms such as artificial intelligence (AI) can be customized for individualized insulin replacement, and potentially replacing the use of exogenous insulin.^46^ Another emerging approach for T1DM therapy is cell‐based insulin delivery, which involves the transplantation of islets or insulin‐producing cells.^45^ Emerging algorithms are improving the capabilities of these integrated hardware and software systems by adjusting interstitial glucose levels to real blood glucose concentrations and predicting insulin doses based on AI‐based interpretations of glucose concentrations. Future trajectory of such technologies may involve monitoring glucose at different body locations, such as the eye or skin, or using optical/vascular techniques to measure blood glucose instead of interstitial glucose, ultimately advancing the field of precision medicine.^45^, ^47^

One compelling example of how precision medicine can transform diabetes care is through the consideration of specific gene single nucleotide polymorphisms (SNPs) like TCF7L2. This gene SNP has been strongly associated with an increased risk of T2DM in certain individuals. In the context of precision medicine, healthcare providers can conduct genetic testing to identify the presence of TCF7L2 SNPs in diabetic patients, providing valuable insights into their genetic predisposition.

For patients carrying TCF7L2 SNPs associated with reduced insulin secretion, precision medicine may involve prescribing medications tailored to enhance insulin secretion, such as incretin‐based therapies like GLP‐1 receptor agonists.^48^ In contrast, patients with different genetic profiles may respond more favorably to alternative treatments. Additionally, precision medicine extends to personalized dietary and lifestyle recommendations, enabling individuals to adopt strategies that best align with their unique genetic predisposition.

Another important gene SNP relevant to precision diabetes care is SLC30A8. This gene SNP is associated with altered insulin secretion from pancreatic beta cells and an increased risk of T2DM in certain individuals.^49^ For patients with SLC30A8 SNPs, precision medicine may involve different treatment considerations, since these individuals may respond differently to glucose‐lowering medications that stimulate insulin secretion, such as sulfonylureas. Instead, they might have a more favorable response to medications that target insulin resistance (e.g., metformin) rather than those that primarily stimulate insulin secretion.

## 5 LATEST TECHNOLOGIES IN PRECISION DIABETES

### 5.1 Transplantation of beta cells

This method primarily aims to restore insulin and glucagon levels, along with other critical secretions, by performing an engraftment of pancreatic islets, especially for patients with insulin‐deficient diabetes, commonly observed in individuals with T1DM. Typically, these islets are harvested from a deceased donor's pancreas and subsequently transferring them into the portal vein for transit through the liver. Several successful transplantations have been reported, resulting in a significant proportion of patients achieving insulin independence.^50^, ^51^, ^52^

In the realm of precision diabetes, the transplantation of beta cells emerges as a promising avenue for individuals, particularly those with T1DM. This approach aligns perfectly with the principles of precision medicine, which emphasize tailoring treatments to each patient's unique characteristics. By meticulously selecting suitable candidates for beta cell transplantation based on a comprehensive analysis of their diabetes profile, encompassing genetic factors, insulin responsiveness, and other individual parameters, healthcare providers can provide highly personalized therapeutic options. Moreover, ongoing research in this field aims to further refine the transplantation process, ensuring that it is optimally suited to the specific needs of each patient.

### 5.2 Nanotechnologies

Recent advancements in nanotechnology have opened up promising avenues in the management of diabetes. One such innovation involves implantable nano‐sensors, which are being developed and studied for CGM. These novel nano‐sensors can be utilized for more precise measurement of blood glucose levels.

Also, the nano‐sensors can be utilized to detect subtle changes in beta‐cell mass, therefore contributing to enhanced diagnosis. Magnetic nanoparticles (MNPs) possess a unique property that renders them excellent contrast agents visible under magnetic resonance imaging (MRI). MNPs have already found applications in various medical conditions, including cardiovascular diseases and cancer, indicating their potential utility in the diagnostic aspect of diabetes. The contrast agents in MNPs can be utilized to monitor beta‐cell function under MRI. The MNP approach can be reliably and effectively used to identify particles and provide noninvasive imaging of beta‐cells.^53^ For example, superparamagnetic iron oxide nanoparticles (SPIONs) are biocompatible which can break down into iron and oxygen. These nanoparticles possess superparamagnetic characteristics that make them a target for magnetic imaging. SPIONs have been developed to track immune cell infiltration and subsequent pancreatitis as an early detection tool for the diagnosis of diabetes. Direct imaging of beta‐cell mass using iron oxide nanoparticles can also be used to track endogenous and exogenous islet cells.^54^

In addition, nanotechnology has also enabled the creation of powerful insulin delivery vehicles that can directly transfer insulin molecules into the bloodstream, bypassing the acidic environment of the stomach and providing an alternative to daily subcutaneous injections.

Nanotechnologies are pivotal in advancing the realm of precision diabetes care, offering finely tuned tools for monitoring and treatment. Implantable nano‐sensors, for instance, enable CGM, facilitating the adjustment of treatment plans based on real‐time data. This real‐time monitoring is inherently personalized, considering each patient's unique glucose variability, which in turn allows healthcare providers to tailor insulin dosages and dietary recommendations with remarkable precision. Furthermore, the application of MNPs for noninvasive imaging of beta‐cell function contributes significantly to early and accurate diagnosis. This technology ensures that interventions are not only timely but also specifically targeted to each patient's unique needs. Additionally, the development of insulin delivery vehicles designed to bypass the stomach's acidic environment offers an exquisitely precise method of insulin administration, custom‐fitted to individual requirements.

## 6 EXPERT OPINION

Precision medicine faces various challenges stemming from the wide range of definitions, which have traditionally been connected to personalized medicine. Regulatory hurdles, patient misconceptions, stakeholders' manipulation of terminology to further their own agenda, and a waning use of the term within the field are all prevalent concerns. Researchers and practitioners in precision diabetes must ensure that such difficulties do not hinder the field's progress and that precision diabetes's promise to enhance health outcomes is fulfilled. Nevertheless, it is challenging to come to a mutual understanding in terms of individualized treatment and management of diabetes.

Diabetes is a chronic autoimmune disease resulting from the irreversible destruction of β‐cells in the body. While advancements in insulin formulations, continuous insulin and glucagon infusion pumps, and CGM systems have been made, they can be costly and offer indefinite relief. Cellular replacement therapy with islet transplantation has shown to be effective in preventing hypoglycemia, correcting hemoglobin A1C levels, and enabling extended periods of insulin independence in some patients. However, the neccesity for lifetime immunosuppressive medicine, susceptibility to infections, malignancies, and nephrotoxicity pose additional challenges that make this treatment unattractive to all but those at high risk of severe hypoglycemia. Additionally, low islet survival after implantation due to innate immune assault, recurrent autoimmune islet destruction, or alloimmune rejection hinder the success of islet transplantations. Strategies focusing on optimizing neovascularization by regulating angiogenesis, reducing inflammation, and lowering oxidative stress can enhance the outcomes of islet transplantation. However, the limited availability of human islets from organ donors renders cellular replacement therapy impractical for every diabetes patient.^55^

To effectively apply precision medicine for diabetes, a variety of data types, including genetic, epidemiological, clinical trial, and medical records, are necessary. However, the available data predominantly come from populations of European ancestry, creating a significant obstacle in implementing tailored therapies in low‐resourced areas and widening health inequities. Additionally, without access to a full range of therapies, technologies, and education, the potential of precision medicine remains unfulfilled.

Due to these challenges, it is unlikely that precision medicine will be implemented widely in the near future. At present, precision diabetes medicine is applicable to a small percentage (2%−3%) of individuals with diabetes, particularly those who have been diagnosed with monogenic diabetes.^56^ Novel models of precision diabetes medicine must be developed, characterized by data‐driven insights, alignment with the biological characteristics of specific populations, and cultural acceptability. For this to happen, more research needs to be prioritized in the most affected countries and regions.

For successful implementation of precision diabetes medicine, collaboration and coordination among multiple stakeholders is crucial, which include research scientists, clinicians, educators, professional organizations, funding agencies, drug regulatory bodies, pharmaceutical companies, policymakers, and diabetes patients. Their efforts should be coordinated across local, national, and international levels to ensure a smooth implementation of precision diabetes medicine.

## AUTHOR CONTRIBUTIONS

**Sharumathy Kannan**: Conceptualization; writing—original draft; writing—review & editing. **Dinesh Kumar Chellappan**: Conceptualization; data curation; formal analysis; investigation; project administration; supervision; validation; writing—review & editing. **Chia Siang Kow**: Conceptualization; data curation; formal analysis; investigation; project administration; supervision; validation; writing—review & editing. **Dinesh Sangarran Ramachandram**: Data curation; formal analysis; investigation; project administration; supervision; validation; writing—review & editing. **Manisha Pandey**: Conceptualization; data curation; formal analysis; investigation; project administration; supervision; validation; writing—review & editing. **Jayashree Mayuren**: Conceptualization; data curation; formal analysis; investigation; project administration; supervision; validation; writing—review & editing. **Kamal Dua**: Writing—review & editing. **Mayuren Candasamy**: Conceptualization; data curation; formal analysis; investigation; project administration; supervision; validation; writing—original draft; writing—review & editing.

## CONFLICT OF INTERESTS STATEMENT

The authors declare no conflict of interest.

## TRANSPARENCY STATEMENT

The lead author, Mayuren Candasamy, affirms that this article is an honest, accurate, and transparent account of the study being reported; that no important aspects of the study have been omitted; and that any discrepancies from the study as planned (and, if relevant, registered) have been explained.

## Data Availability

Data sharing is not applicable to this article as no new data were created or analyzed in this study.
